# Communication on Safe Caregiving between Community Nurse Case Managers and Family Caregivers

**DOI:** 10.3390/healthcare9020205

**Published:** 2021-02-14

**Authors:** María Eulalia Macías-Colorado, Margarita Rodríguez-Pérez, María Jesús Rojas-Ocaña, Cristina Teresa-Morales

**Affiliations:** 1 Training, Research, and Quality Unit, Huelva-Costa and Condado-Campiña Primary Care District, Andalusian Health Service, 21004 Huelva, Spain; mariae.macias.sspa@juntadeandalucia.es; 2 Nursing Department, Faculty of Nursing, University of Huelva, 21007 Huelva, Spain; mariaj.rojas@denf.uhu.es (M.J.R.-O.); cristina.teresa@denf.uhu.es (C.T.-M.)

**Keywords:** family caregivers, home care, patient safety, risk management, health communication

## Abstract

Dependent elderly individuals are usually cared for at home by untrained family members who are unaware of the risks involved. In this setting, communication on safe caregiving is key. The aim of this study is to describe the factors influencing the process followed by community nurse case managers to provide communication on safe caregiving to family members caring for dependent elderly individuals. A phenomenological study, by focus group, was done in urban healthcare facilities. Key informants were seven community nurses, case managers with more than 12 years’ experience. We did a thematic analysis and we identified the units of meaning to which the most relevant discourses were assigned. The concepts expressed were grouped until subcategories were formed, which were then condensed into categories. Four categories of analysis emerged: communication-related aspects; professional skills of nurse case managers; communication on safety and the caregiving role. To planner interventions, for the prevention of adverse events at home, is essential to consider these aspects: nurses’ professional communication skills, factors inherent to safe caregiving, the characteristics of the home where care is provided, the personal and family circumstances of the caregiver, and whether or not the caregiver’s role has been assumed by the family caregivers.

## 1. Introduction

It is increasingly common for patients facing a process of dependency to choose to remain at home under the care of their family members. This is a difficult time for the individual taking on the caregiving role, the family caregiver (FC), who is often insecure and fearful and needs information and support from healthcare professionals [1]. Community nurse case managers (CNCMs) (the figure of the nurse case manager (subsequently CNCM)) was created in Andalusia, Spain, in 2002 pursuant to Decree 137/2002 of 30 April on Support for Andalusian Families. Section VII covers support measures for the elderly and/or disabled, including the promotion and improvement of home care to ensure that these individuals can remain at home for as long as possible) are the first professionals to come in contact with FCs when a sudden change in patients’ health occurs [2]. The home setting is the specific physical environment where caregiving takes place, where the experiences of FCs and the individuals under their care are shaped, and where CNCMs play a key role in ensuring that individuals can continue to be cared for at home, an environment that reflects their beliefs and culture. The home can also be a risky setting for care provision [3], which makes it necessary for CNCMs, who are aware of the potential risks involved in home care, to consider patients and caregivers responsible for key elements of safety [4,5]. A review of studies published over the past decade indicated that the most common risks and adverse events (AEs) resulting from home care are falls and injuries; urinary tract infections with and without catheterisation; ulcer and wound infections; pressure ulcers; medication errors; malnutrition; non-compliance with the therapeutic regimen; isolation; and psychosocial, mental, and behavioural issues [6,7,8,9,10,11,12]. A number of studies argue that home care safety risks should include FC related risks, such as caregiver role strain [8] and the erosion of the home as a safe space for the family [11]. These risks can be exacerbated by errors in the transmission of information and by other issues associated with the communication process [13]. FCs tend to depend solely on themselves to identify the risks associated with the care they provide. FCs carry out certain care interventions without understanding the risks involved and without taking appropriate safety precautions, which could have serious implications. Studies conducted in other countries identify various degrees of causality between AEs and home care providers’ performance, ranging from probably related (50% of the AEs identified) to almost certainly related (27.9%) [10]. For this reason, FCs and family members often request information about fundamental aspects relating to care, safety, and the risks involved [13], which must be provided by CNCMs. In order to minimise these risks, the APEAS report—a study on patient safety in primary healthcare conducted in Spain—recommends that communication should be improved in primary healthcare (PH) to ensure patient safety [14]. CNCMs possess the skills needed to identify and prevent risks, improving the safety of home care delivery [1]. Therefore, a good relationship between CNCMs and FCs is essential to ensure that FCs fully understand how care should be provided and are truly aware of the risks involved. This is why communication between the two is commonly emphasised as the cornerstone of safety [15]. Consequently, it is of paramount importance to identify the factors influencing communication on safety between CNCMs and FCs [13,16].

The communication model used in transitions of care has been studied from the perspective of patients being transferred from one hospital department to another, from inpatient care to PH and, to a lesser extent, from inpatient care to their homes [3]. Further studies are needed to assess the experiences of patients and caregivers in the transfer of information on home care safety [17]. As a result, the aim of this study is to describe, from a phenomenological perspective, the factors influencing the process followed by CNCMs to provide communication on safe caregiving to family members caring for dependent elderly individuals.

## 2. Materials and Methods

This is a qualitative phenomenological study with a focus group session involving seven CNCMs from seven urban healthcare facilities in the city of Huelva who had worked as professional CNCMs for more than one year at the time of the study. The skills, performance, and professional role of these advanced practice home care nurses made them key informants in this research. The dimensions studied were as follows:

1. The social and occupational profiles of the participating CNCMs, including data on sex, age, years of professional experience in primary healthcare, years as a CNCM, and up-to-date patient safety training. Data were collected using a Google Forms© questionnaire.
2. Components of communication on safe caregiving. In order to detail these components, a literature review was conducted using the following databases: PubMed, CUIDEN, CINAHL, Cochrane, SciELO, and Epistemonikos, and the following MeSH descriptors: “communication”, “caregiver”, “home nursing”, “patient safety”, “advanced practice nursing”, as well as their equivalent DeCS terms. From the results obtained, the following data were selected: the types, styles, and channels of communication used to provide information on recommended care; the methods used to verify that the information has been understood; safety-related nursing interventions; the transfer of care-related information to other nurses; and the notification to AEs identified in patients’ homes. Based on this information, a script with questions was prepared for the focus group session, which has been attached as an appendix.

In order to make it easier for CNCMs to express their views on how they provided communication on safe caregiving, a focus group was held for data collection, since focus group session promote discussion and prompt participants to interact with one another while allowing researchers to explore and delve deeper into participants’ perceptions, opinions, thoughts, knowledge, attitudes, experiences, and behaviours [18,19]. According to the scientific literature, the number of participants was sufficient for a focus group session, especially considering that the participants were key informants [18,19].

The CNCMs were contacted by telephone to arrange an individual meeting to inform them about the study. The focus group session lasted 65 min and was held in a comfortable, accessible location at an agreed time. It was audio- and video-recorded with the consent of the participants. Two researchers moderated the session and noted their observations in a field notebook.

The focus group session was transcribed verbatim, and an initial reading was conducted. The researchers conducted several readings, individually and intentionally. Subsequently, following Giorgi’s guidelines [20], the data underwent thematic analysis that began by identifying the units of analysis to which the most significant discourses were assigned. The concepts expressed were grouped together by comparing and contrasting them systematically until subcategories were formed, which were then condensed into categories [21,22]. To ensure the reliability of the study through triangulation, the information was analysed by three researchers independently, who took into account the principles of transferability, consistency, reflexivity, and relevance [18,19]. ATLAS.ti 8.0© software, Scientific Software Development Gmb (Berlin, Germany) was used for qualitative analysis.

The protocol followed in this study, which established ways to preserve and protect participants’ confidentiality and anonymity in accordance with current Spanish legislation, was approved by the Huelva Research Ethics Committee. Participants collaborated voluntarily and were free to withdraw from the study at any time without giving a reason. Once any questions had been answered, participants signed the informed consent form.

## 3. Results

The seven CNCMs were predominantly women, aged between 40 and 50 years old, with an average of 26 years’ experience as primary care nurses. Sixty-six percent of them had more than 12 years’ experience as a CNCM. Eighty-three point three percent had received patient safety training, although only half had received it in the previous year. In their workplaces, patient safety training sessions were held 1–2 times per year, addressing aspects such as AEs, hand washing, safe procedures, reporting of incidents, and administration of medication. Participants had received more training on skin protection and medication management than on communication and nutrition.

Four categories related to the communication process were identified: communication-related aspects, professional skills of CNCMs, safety in communication, and the caregiving role. A total of 21 subcategories were established (Table 1). Most of the discourses of the CNCMs reflected a holistic perspective, making it difficult to analyse each factor separately. The interrelationships between the various factors hindered the ability to associate each discourse with a specific subcategory, as many of them could have been included in several subcategories.

Table 1: The interrelationships between categories, codes and subcategories are shown in this table. The units of meanings have been identified based on the most significant discourses, which will be presented in the next tables. The concepts expressed were grouped together until 21 subcategories were formed, which were then condensed into 4 categories

For the CNCMs, the factors influencing communication on safe caregiving are related to factors intrinsic to safety itself, as well as to communication skills that professionals must possess.

*E1: “I think adverse events occur for [two] different reasons. Firstly, non-communication and, secondly, incomplete or inadequate communication. But primarily due to non-communication, and I think that has always had to do with culture.”*

The factors influencing communication on safe caregiving are also related to the FC’s family and personal circumstances, as well as to the environment in which caregiving takes place. Professional skills, communication skills, and the different components of the role of FC also influenced communication on safe caregiving. Among the factors inherent to the communication process, the CNCMs stressed that they had to adjust their communication to the circumstances of the patient or the FC, which were shaped in turn by the life stage of the family and their ability to explain caregiving tasks depending on their needs. To ensure safer communication, CNCMs considered it essential to involve patients and FCs in the communication process (Table 2).

Table 2: In this table, the most relevant discourses, associated with the CAT-C, Communication-related aspect, are presented. They are located in the subcategories to which they have given meaning.

Professional skills were the key to safety for the CNCMs. They believed that a safety-centred approach to caregiving would be impossible without these skills, and they placed particular emphasis on safe communication, accessibility, and the bonds they build with FCs. They recognised the importance of intervention and the need to possess the necessary knowledge (Table 3).

Table 3: In this table, we can see the most significant discourses to understand the meaning of the subcategories associated with the CAT-P, Professional Skills of CNCMs.

The CNCMs mentioned the safety aspects involved in communicating information on care. They explained that they provided information on care but found it difficult to identify the risks they needed to communicate to caregivers and too the community nurses, to whom they detailed the circumstances of the patient and caregiver, the interventions they had implemented, and any other aspects to be considered. However, they failed to focus on safety, verify whether information had been properly understood, or perform a follow-up. They valued reinforcing the importance of caregiving among FCs and said that they used verbal and non-verbal communication, finding that written documents were less helpful.

The use of new technologies was debated by the CNCMs, with a mix of opinions both for and against. They all agreed that the use of new technologies could support written information and improve communication and caregiving. The problem lay in the fact that they did not have work mobile phones allowing them to use apps and other new technologies and had to use their personal mobile phones instead, which they did not feel comfortable doing. The mechanisation of the communication process was viewed positively, as professional experience facilitated communication, but information was automatically repeated without the need for reflection (Table 4).

Table 4: This table, called “Category S: Safety in communication”, includes the CNCMs’ discourses relatedto the verification and understanding to the information on care, for the FCs.

The CNCMs considered the factors associated with the caregiver and their family environment to be crucial for safety. These factors permeated their discourses and were interrelated within the same category. One essential factor was the need for the caregiver to accept and assume their role. Accepting the caregiving role refers to the FC deciding—of their own accord or on behalf of their relatives—to become the primary caregiver, i.e., they approve that role. However, assuming the caregiver’s role means internalising that responsibility and executing it using the knowledge and resources available. The CNCMs concurred that when caregivers assumed their role, they provided safer care and were more willing to communicate. Another factor that was highlighted as negative for safety was the presence of several caregivers who had not assumed the caregiver’s role. This blurred responsibilities for caregiving and affected the transfer of information, compromising safety. The CNCMs linked family support to safety in two respects: recognition by other family members of the FC’s commitment and dedication, and assistance with caregiving tasks from other family members to allow the caregiver to have free time. The family’s income level is another important factor for caregiving, determining whether or not it is possible to purchase caregiving aids such as technical aids or home adaptations that CNCMs associate with safe patient mobility and reduced physical risks at home (Table 5).

Table 5: In this table, we show the aspect associated with accepting the caregiver’s role, assuming the responsibility for caregiving, and some other FCs’ and family’s characteristics, which are formatted this category, called “The caregiving role”.

## 4. Discussion

The CNCMs had a uniformly experienced professional profile, including training in safety strategies.

Communication was a fundamental aspect of nurses’ professional skills and was considered critical when transferring caregiving to the home setting. The CNCMs agreed that factors inherent to the communication process itself and involving patients and FCs in the care plan are also important. As other studies have shown, adapting the information provided favours more effective communication [23]. However, participants acknowledged that the communication process is almost exclusively verbal, with little support from other tools, although they have systematised the transfer of information. As in other studies [13,23], FCs demanded additional information in writing from CNCMs to support recommendations for care. However, information is often only provided verbally, rendering it critical to patient safety.

A number of participants reported not using new technologies because they lacked the necessary means to do so. This is an important aspect to consider, since the use of new technologies can contribute to improving and quickening communication and effective caregiving, as well as fostering a closer relationship between CNCMs and FCs [17,23,24].

The CNCMs acknowledged that they do not expressly verify that the information they provide is properly understood of information. Although a safety checklist of dangerous situations at home has been proposed, it does not include the transfer of caregiving to FCs [3], and few studies have looked into this. Therefore, there is a need to design, validate, and implement tools that allow CNCMs to verify that the information on safe caregiving that they provide is properly understood in a standardised manner.

Factors relating to communication on safe caregiving were linked to professional skills regarding safety knowledge and communication skills. The communication process is also shaped by the personal and family circumstances of the FC, which have been addressed in studies on the need to establish good communication in the early stages of caregiving [1]. Participants noted that FCs are more open and willing to communicate once they have assumed the caregiver’s role, which involves accepting responsibility for caregiving and learning how to provide care safely. Communication becomes more difficult when caregiving tasks are shared by several FCs, resulting in blurred responsibilities and increased risks.

Participants highlighted the influence of the context where caregiving takes place (family environment, income level, and the physical environment at home) on the safety of the care provided, which was also shown to distort communication, in line with the findings of a previous study [3]. In this context, when transferring patients to their homes and in the absence of safety strategies in this area, CNCMs are unable to implement these strategies or comply with the safety guidelines laid down for other healthcare areas.

A limitation of this study has been to do only one focal group; however, this focal group was formed by all the CNCMs from the basic health zones of the city of Huelva. This limitation offers the possibility of further research in other health zones, which will complement our contributions and allow us to establish future safe home care strategies.

## 5. Conclusions

Home care provided by family members is fraught with risks, and the transfer of information between CNCMs and FCs is a particular problem. When planning interventions for the prevention of AEs at home, it is essential to consider the following aspects: nurses’ professional communication skills, factors inherent to safe caregiving, the characteristics of the home where care is provided, the personal and family circumstances of the caregiver, and whether or not the caregiver’s role has been assumed by the FC. In addition, communication on safe caregiving becomes more difficult when caregiving tasks are shared by several FCs, resulting in increased risks.

Avenues for improving the safety of home care may include adapting the information provided to FCs so that it is easier to understand and verifying that the information is properly understood by using instruments to standardise this verification. It would also be helpful to incorporate tools based on new technologies.

Finally, studying perceptions of the construct of safety by CNCMs and FCs would facilitate the planning of safety strategies similar to those applied in hospital and PH settings, without overlooking the specific environment where care is delivered and experienced.

## Figures and Tables

**Table 1:** Interrelationships between categories, codes, and subcategories.

Category	Code	Subcategory
Communication-related aspects	CAT-C	C1-CNCMs’ ability to adapt to the circumstances of the patient and the caregiver
		C2-Including the patient and caregiver in the communication process
		C3-Using verbal communication
		C4-Mechanisation of the communication process
		C5-Use of other forms of communication: writing, ICT, etc.
Professional skills of CNCMs	CAT-P	P1-Knowledge, skills, and attitudes of CNCMs
		P2-Evaluation of outcomes
		P3-Building a personal bond with caregivers
		P4-Being accessible to caregivers
Safety in communication	CAT-S	S1-General information on care
		S2-Information on caregiving risks
		S3-Information on safety measures
		S4-Transfer of information to another professional
		S5-Transfer of information to the caregiver
		S6-Ensuring that the information has been understood
		S7-Reinforcing the caregiving role
The caregiving role	CAT-R	R1-Accepting the caregiving role by the caregiver
		R2-Assuming responsibility for caregiving
		R3-Family support and family income level
		R4-Distribution of caregiving tasks among several family members
		R5-Competence and capacity for caregiving

**Table 2:** Category C: Communication-related aspects. (The figure of the nurse case manager (subsequently CNCM) was created in Andalusia, Spain, in 2002 pursuant to Decree 137/2002 of 30 April on Support for Andalusian Families. Section VII covers support measures for the elderly and/or disabled, including the promotion and improvement of home care to ensure that these individuals can remain at home for as long as possible).

Category	Communication-Related Aspects
Subcategories	Associated Discourses
C1-CNCMs’ ability to adapt to the circumstances of the patient and the caregiver	E1-“Yeah, yeah, they give you your space. That’s very important, because when they give you your space, they are showing you the type of relationship they [want to] have with you (...) they may want a closer relationship or a more distant one.” E2-“Respect always prevails (...) the home is always a totally private place, you know, privacy.” E3-Indeed, because she’s so overwhelmed with this new situation, she doesn’t know where to start, so I try to give her small pieces of information (...) to help them sort things out.
C2-Including the patient and caregiver in the communication process	E3-“When the patient is conscious and you address the caregiver, the feeling that [the patient] has (...) [is] that we’re talking about him but he’s being left out. So it’s better (…).” E5-“Helping the two parties involved [in care] cope with the situation.”
C3-Using verbal communication	E1-“It’s more to do [with verbal communication] because [the patient] is unable to communicate or explain themself. If [the patient] is unable to explain themself (…).” E6-“The posture, the tone (...) everything (...).”
C4-Mechanisation of the communication process	E4-“I think we have corrupted [the communication process], after all these years (…) we have corrupted it, because you just know the moment you walk into a house (…) you just see it perfectly (…) [and you think] ‘I know [the caregiver’s] capabilities.’”
C5-Use of other forms of communication: writing, ICT, etc.	E7-“Depending on the type of information, we use Facebook (…), because many people don’t come to the workshops, so we usually offer multidisciplinary training through Facebook (…). I also have two email accounts, two WhatsApp accounts (...)”. E4-“My personal [phone] number is my personal [phone] number, and no [patient or caregiver] gets it, I’m sorry.” E3-“I just give my mobile phone number. They usually call you because (…) it’s the work phone number and that’s it.”

**Table 3:** Category P: Professional skills of CNCMs.

Category	Professional Skills of Cncms
Subcategories	Associated Discourses
P1-Knowledge, skills, and attitudes of CNCMs	E1-“It’s just that nurses don’t take responsibility for caregiving and sometimes people... well, we may be dealing with adverse events sometimes, but other times it’s sheer ignorance, because we need to take control of the culture once and for all, I mean, what is my responsibility is my responsibility.”
P2-Evaluation of outcomes	E2-“I’ll just go and do my job, just as the nurse in charge of the patient will keep on doing their job and has other competences. I can’t even think of doing everything because I probably won’t know how to do it all in the first place.”
P3-Building a personal bond with caregivers	E3-“In cases of highly dependent patients or similar situations, I try to always be in the background, okay? I don’t give up on the case, I try to always be there as a point of reference, I keep doing the calls.”
P4-Being accessible to caregivers	E5-“You’re also showing yourself to be accessible to them, offering them a type of relationship that may be a little more intimate (…).” E7-“(…) I always tell them: ‘this is like a long-distance race. You don’t have to worry about us, I’ll always be there for you. Call me whenever you need me and we’ll address your concerns. This is only the beginning, so we’ll take it step by step’, you know?”

**Table 4:** Category S: Safety in communication.

Category	Safety in Communication
Subcategories	Associated Discourses
S1-General information on care	E1-“Transfer models, inhaler use, managing public aid, of course! (...) and a bit of device handling (…)”.
S2-Information on caregiving risks	E4-“I tell them ‘get the bag out for me please, let’s tidy up all this and remove these bags (…) in bundles’. They’re unbelievable (…).” E2-“Well, nobody has explained it to them, right? Or maybe someone has, or we have and we’ve taken it as a given, I don’t know.” E4-“Let’s go over the medications. ‘Oh! Is this not the same as this one? Or this one? (...) I thought they were the same. They changed my patch and now they’re the same colour’ and you go ‘Oh!’”. E6-“I think adverse events occur for [two] different reasons. Firstly, non-communication and, secondly, incomplete or inadequate communication. But primarily due to non-communication, and I think that has always had to do with culture.”
S3-Information on safety measures	E7-“Environmental control measures always (...) you’ll have to buy another one, you’ll have to remove this cable, and this chair goes out, too.” E2-“(…) for example, checking the first-aid kit. Is that [a] patient safety [measure]?”
S4-Transfer of information to another professional	E1-“(…) well, I just tell the nurse ‘this is what I have worked on and I won’t do that follow-up check.’ I tell that to their primary care nurse so she can continue with the follow-up.” E2-“You can do that in a jiffy. You just have to take the person’s data and give them to the nurse.” E4-“(…) Word of mouth is always crucial (…) either I tell them myself or I won’t be certain.” E5-“Because we are often biased by what matters most to each of us. Maybe she cares more about it than someone else does, and I do care (…).”
S5-Transfer of information to the caregiver	E3-“Indeed, because she’s so overwhelmed with this new situation, she doesn’t know where to start, so I try to give her small pieces of information (...) [gesture of giving information in small doses] to help them sort things out.” E1-“(…) I don’t have any specific procedure for giving out information. I always ask ‘Was that clear? Do you have any questions? Call me if anything comes up’ and things like that (…).”
S6-Ensuring that the information has been understood	E4-“I ask them to demonstrate and repeat [what I have just taught them].” E1-“I don’t verify it [repeats it three times] (...) I just go and give them the whole lecture and then we recap: ‘This, this, and that. Was that clear? Yes. Do you have any questions? No. Okay then.’”
S7-Reinforcing the caregiving role	E1-“Come on, let’s see how you do it and so on, because fear is only fear (…) this happened to me so suddenly, I don’t even know where to start, but now that I’m out of the hospital and all this is my responsibility, I want to learn, I don’t want to cause any harm, I want to know that I’m doing this properly.” E2-“If there’s an appointed caregiver, he or she should feel that [responsibility] (…). That’s necessary for both the caregiver and the patient.”

**Table 5:** Category R: The caregiving role.

Category	The Caregiving Role
Subcategories	Associated Discourses
R1-Accepting the caregiving role by the caregiver	E3-“So no one wants to be labelled as the primary caregiver, as we call it, because that means that he or she is the primary caregiver and the others are the little soldiers who duck out (…).”
R2-Assuming responsibility for caregiving	E1-“There’s one thing we don’t do, but we should do, and that is ask who’s going to be the primary caregiver and why, who’s going to be the primary caregiver and why (she repeats). Why is that person going to be the primary caregiver? Because they chose to? Because it was their turn or what? (…).” E2-“And it’s not only about the harm that that person may cause while using their skills or a technique, but also about the harm, the suffering, the discomfort they may experience because their life will never be the same, coping (…).”
R3-Family support and family income level	E4-“When a new situation arises suddenly, so abruptly, causing [someone to be] highly dependent, highly disabled, people say ‘I want you to organise my life, to tell me what’s going on, because I don’t know what’s going on, I don’t know if (…)’ and I tell them ‘Now, stop, stop. How many of you will be able to provide care? What’s the family situation like? Now, let’s organise ourselves and see what we can count on, so we’ll know where we have to start.’” E7-“(…) when there’s no [human] support, they want to compensate for it with equipment, but it’s about [human] support, not equipment. ‘But she wants a [mechanical] loader and a crane.’ ‘I know, but, who’s going to use them? Your mother, on her own? Is she going to operate them? Because there won’t be anyone here [to help].’ And that’s not an option. First [things first]: who will be there [to help]?”
R4-Distribution of caregiving tasks among several family members	E4-“And when there are several caregivers who disagree and the patient’s safety is at risk but all three are there, aren’t they? Aren’t all three of us the caregivers? All three of us are. (…) Everyone’s safety goes pear-shaped just because nobody takes responsibility.”
R5-Competence and capacity for caregiving	E2-“Of course, that’s working a bit with their relatives. You can work a bit on their roles, on the confrontations in the family (...) but usually you can’t impose [anything on them].”

## Data Availability

Not applicable.
